# A comprehensive review of molecular optimization in artificial intelligence‐based drug discovery

**DOI:** 10.1002/qub2.30

**Published:** 2024-02-12

**Authors:** Yuhang Xia, Yongkang Wang, Zhiwei Wang, Wen Zhang

**Affiliations:** ^1^ School of Information Huazhong Agricultural University Wuhan China

**Keywords:** artificial intelligence, drug discovery, molecular optimization

## Abstract

Drug discovery is aimed to design novel molecules with specific chemical properties for the treatment of targeting diseases. Generally, molecular optimization is one important step in drug discovery, which optimizes the physical and chemical properties of a molecule. Currently, artificial intelligence techniques have shown excellent success in drug discovery, which has emerged as a new strategy to address the challenges of drug design including molecular optimization, and drastically reduce the costs and time for drug discovery. We review the latest advances of molecular optimization in artificial intelligence‐based drug discovery, including data resources, molecular properties, optimization methodologies, and assessment criteria for molecular optimization. Specifically, we classify the optimization methodologies into molecular mapping‐based, molecular distribution matching‐based, and guided search‐based methods, respectively, and discuss the principles of these methods as well as their pros and cons. Moreover, we highlight the current challenges in molecular optimization and offer a variety of perspectives, including interpretability, multidimensional optimization, and model generalization, on potential new lines of research to pursue in future. This study provides a comprehensive review of molecular optimization in artificial intelligence‐based drug discovery, which points out the challenges as well as the new prospects. This review will guide researchers who are interested in artificial intelligence molecular optimization.

## 1 INTRODUCTION

Drug discovery is an expensive, risky, and complex procedure with a long development cycle. The successful development of an innovative drug will cost between 0.8 and 1.5 billion dollars and take 10–15 years [1, 2]. Annually, around 90% of drugs assessed by the Food and Drug Administration (FDA) are rejected and cannot be utilized for medicinal purposes [3]. Therefore, it is essential to consider efficient techniques to combat the rising cost and hasten the discovery process.

In drug discovery, discovering novel drug candidates for preclinical trials can be summarized into five steps [4, 5]. The first step is the identification of drug targets. The second step is screening the hit compounds with on‐target activity from the molecular databases. Then, based on the initial validation for hit compounds, the drug‐likeness compounds (lead compounds) are collected. After that, the molecular optimization step is implemented to optimize the druggability of lead compounds. Finally, the safety of the drug candidates is preliminarily validated before the preclinical studies.

In the past decades, computer‐aided drug design (CADD) has made use of domain knowledge in the chemical, pharmacological, and biological fields for drug discovery, and high‐throughput screening (HTS) has considerably accelerated drug discovery [6, 7]. Due to the rapid progress in processing power and increasing amounts of cheminformatics data, artificial intelligence (AI)‐based methods have recently been flourishing and brought new opportunities and solutions for drug discovery [8, 9, 10, 11, 12, 13, 14, 15]. The above‐mentioned information techniques are greatly involved in several key steps of drug discovery before clinical trials, including drug target identification, hit compound screening, molecular optimization, and safety evaluation of drug candidates.

It is notable that the structure of drug candidates determines the drug properties and clinical effects, while the screened candidates typically have certain pharmacological flaws showing obstruction in clinical application [16, 17]. Thus, molecular optimization is one of the greatest challenges in drug discovery [18, 19], aiming to enhance the physical and chemical properties of a molecule, such as solubility, bioavailability, and selectivity [20]. Since the structures of molecules can determine their properties, researchers constructed the quantitative structure–activity relationship (QSAR) that describes a mathematical relationship between quantitative representations of molecule structures and the activity or property of interest [21]. Traditional computational methods for molecular optimization consider a molecule as a set of fragments, which are responsible for different functions, and then modify (add, delete, or replace) fragments of a molecule to enhance its properties according to QSAR. As there are a great number of molecular fragments, the combination of fragments will form a huge chemical space, and finding the desirable molecules from the chemical space is an NP‐hard task. To improve the efficiency of molecular optimization, researchers developed AI‐based molecular optimization methods by using natural language processing (NLP), swarm intelligence, reinforcement learning, generative adversarial network, etc.

In this review, we concentrate on the development of artificial intelligence‐based molecular optimization. Firstly, we outline the datasets for molecular optimization and approaches to molecular representation. Then, we introduce the important molecule properties as well as three types of mainstream AI‐based molecular optimization methods, including molecular mapping, molecular distribution matching, and guided search‐based methods. After that, we summarize the assessment criteria for optimization models as well as the benefits and drawbacks of existing methods. Finally, we discuss the challenges of molecular optimization and highlight the cutting‐edge directions for future optimization research, that is, model interpretability, multi‐property optimization, and model transferability.

## 2 DATASETS

### 2.1 Data sources for drug discovery

There are several public databases about drug molecules, including ZINC [22], ChEMBL [23], and DrugBank [24]. ZINC is a free database of 2.3E + 08 compounds constructed by the researchers from the Department of Pharmaceutical Chemistry at the University of California, San Francisco (UCSF), providing the log *p* value of molecules by using Molinspiration software [25]. ChEMBL is a large, open‐access drug discovery database with 2.4E + 06 compounds developed by the European Bioinformatics Institute (EBI), aiming to capture the medicinal chemistry data across the drug discovery process [23], which is calculated by RDKit [26] and Chemistry Development Kit (CDK) [27]. DrugBank is an online database created by Dr. David Wishart’s lab at the University of Alberta, containing the experimental and predicted properties on over 5E+05 drugs and drug targets [26]. The molecules and their properties provided by the above databases are listed in Table 1.

**TABLE 1:** Public databases and datasets for drug discovery.

Databases/Datasets	Size (number of molecules)	Properties	Source of properties
ZINC	2.3E + 08	log P	Molinspiration [25]
ChEMBL	2.4E + 06	ADMET, QED, log P, log D, bioactivity	RDKit [26], CDK [27]
DrugBank	5E + 05	ADMET, log P, log S, water solubility	Chemaxon [34], ALOGPS [35], bio‐experiment
QM9	1.34E + 05	Energy, enthalpy	B3LYP/6‐31G(2df,p)
Tox21	1.37E + 04	Toxicity	qHTs [33]
BACE	1.5E + 03	Bioactivity (BACE‐1)	Bio‐experiment
BBBP	2E + 03	Permeability	Bio‐experiment
Lipo	4.2E + 03	log D	Bio‐experiment

The researchers have also constructed several public datasets such as QM9 [28], Tox21 [29], BACE [30], BBBP [31], and Lipo [32]. QM9 is a dataset with 1.34E + 05 molecules built by Ramakrishnan et al. [28], calculating the energy and enthalpies of molecules by the quantum chemistry computational method named B3LYP/6‐31G(2df, p). Tox21 is a dataset comprising 1.37E + 04 compounds compiled by the National Institutes of Health, the Environmental Protection Agency, and the Food and Drug Administration in America, containing toxicity measurements for six common targets of xenobiotic toxicity (the liver, blood, kidney, nerve, lung, and skin) [29], gained from the quantitative high‐throughput screening (qHTs) [33]. BACE is a dataset with 1.5E + 03 compounds created by Subramanian et al. [30], containing molecular bioactivity against a set of inhibitors of human b‐secretase 1 (BACE‐1), selected from the bio‐experiment. BBBP is a collection of 2E+03 molecules from Martins et al. [31], including binary labels for compounds on their permeability properties, gained from the bio‐experiment. Lipo is a sub‐dataset with 4.2E + 03 molecules from the ChEMBL database, providing the log D of compounds [32], selected from the bio‐experiment. Table 1 shows the molecules and their properties provided by the above datasets.

### 2.2 Molecular properties for molecular optimization

As shown in Table 2, there are four types of molecular properties of great interest in drug discovery: pharmacokinetic properties, medicinal chemistry properties, physicochemical properties, and pharmacological properties [36, 37], and researchers usually optimize some properties of molecules, including Absorption(A), Distribution(D), Metabolism(M), Excretion(E), Toxicity(T), which are collectively called ADMET, and Drug likeness (QED), octanol‐water partition coefficient (log P), octanol‐water distribution coefficient (log D), molecular bioactivity against drug targets (e.g., DRD2, GSK3β, and JNK3), as well as selectivity. ADMET is a set of pharmacokinetic properties, which provides insight into how a drug interacts with the human body. QED is a kind of medicinal chemistry property, measuring how likely a molecule is to be a potential drug candidate. SA represents the ease of molecular synthesis respectively. log P and log D measure the solubility of a compound. DRD2 is the main target of most psychiatric drugs for schizophrenia and Parkinson’s disease; GSK3β and JNK3 are drug targets for Alzheimer’s disease. Selectivity refers to the degree to which a drug interacts specifically with its intended target in the body.

**TABLE 2:** Common molecular properties for molecular optimization.

Types	Properties
Pharmacokinetic	Absorption(A), Distribution(D), Metabolism(M), Excretion(E), and Toxicity(T).
Medicinal chemistry	Drug likeness (QED) and synthetic accessibility (SA).
Physicochemical	Octanol‐water partition coefficient (log P) and octanol‐water distribution coefficient (log D).
Pharmacological	Bioactivity and selectivity.

Notably, the pharmacological properties are usually evaluated by a property predictor, and the other properties are usually evaluated by RDKit. In addition, it is fundamental for molecular optimization to ensure the structural similarity of molecules before and after optimization, which is usually calculated by RDKit.

### 2.3 Datasets for molecular optimization

In molecular optimization, researchers usually collect their own data from the above databases according to certain optimization tasks. According to the study [38], two molecules that have small differences between consecutive atoms but huge differences between properties can be considered as a matched molecular pair (MMP), and the molecule with poor properties in an MMP is named the source molecule, the molecule with desired properties is named the target molecule. For example, He et al. [39] compiled a dataset with 9,927,876 MMPs and their ADMET properties from ChEMBL. Since it is difficult to collect the MMPs related to all optimized properties, researchers try to collect two sets of molecules with huge differences between properties, which we name as matched molecular set (MMS), and the molecular set with poor properties in an MMS is named as source molecular set, the molecular set with desired properties is named as target molecular set. For example, Barshatski et al. [40] collected a matched molecular set with 1897 molecules and their bioactivity to DRD2 from DrugBank. Unlike MMPs and MMS, some researchers directly collect molecules and their properties for optimization. For example, Madhawa et al. [11] collected 2 datasets with 2.5E + 05 and 1.34E + 05 molecules from ZINC and QM9 respectively, directly for the optimization of QED and log P. Through the above three strategies of data collection, researchers can also create their own datasets for molecular optimization.

### 2.4 Molecular representations

It is a fundamental challenge to represent molecules in a form that computers can understand easily once trustworthy data has been gathered. In molecular optimization, molecules are usually represented by strings and graphs.

#### 2.4.1 String‐based representation

**FIGURE 1:**
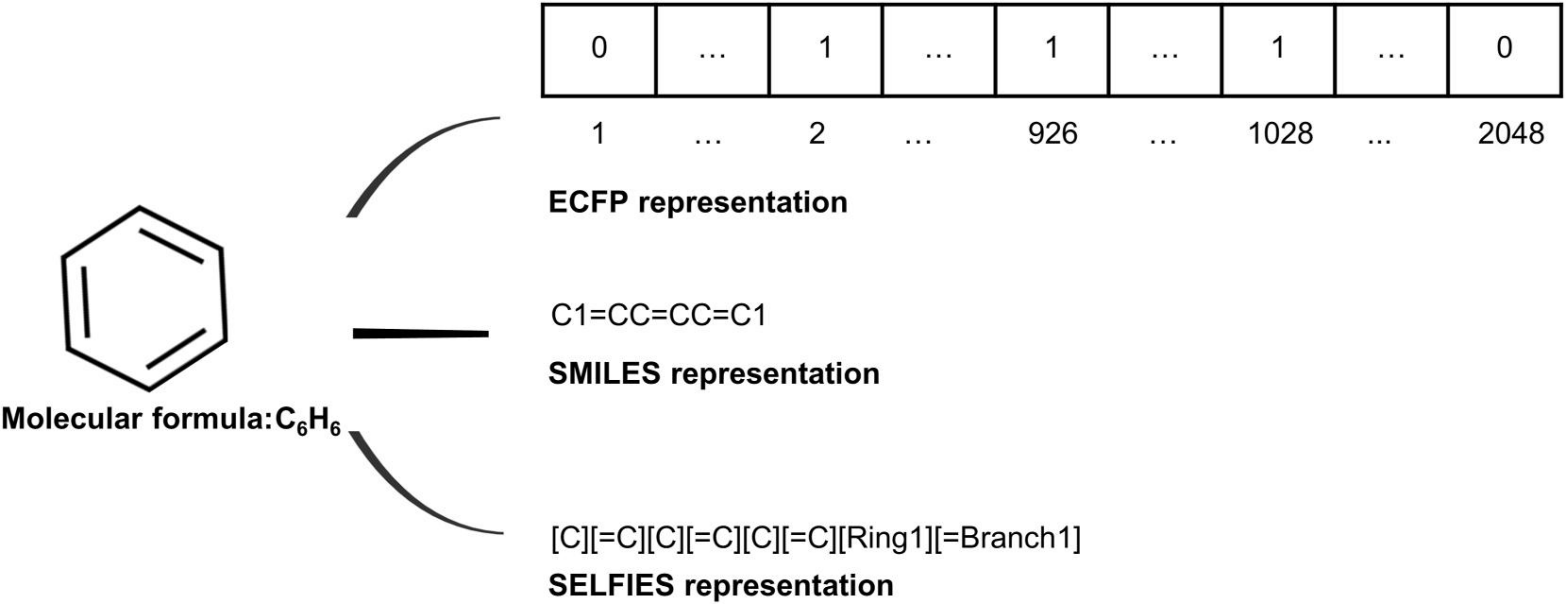
The molecule C_6_H_6_ is represented by three kinds of string‐based representations.

As a type of string‐based representation, molecular fingerprints are high‐dimensional bit vectors of molecular chemical structures where each bit indicates whether a structure exists, such as substructure key‐based fingerprints, topological or path‐based fingerprints, and circular fingerprints. Although molecular fingerprints have the advantage of containing structural information, they cannot be reconverted into a unique molecule necessarily. Simplified molecular input line system (SMILES) is a classic linear representation for molecules with short ASCII strings [41]. Specifically, SMILES encodes atoms into letters, bonds into symbols, and branches into parentheses. SMILES are space‐saving and can correspond to a unique molecule, but a change of a single character may result in an invalid molecule, which does not meet the basic chemical rules. SELF‐referencing embedded strings (SELFIES) [42] is a string representation in which every symbol is interpreted as a rule vector. SELFIES can avoid grammatical errors by enforcing chemical validity rules in a formal syntax table to ensure 100% molecular validity. As shown in Figure 1, a molecule formulated as C_6_H_6_ can be represented by the above three kinds of string‐based representations.

#### 2.4.2 Graph‐based representation

**FIGURE 2:**
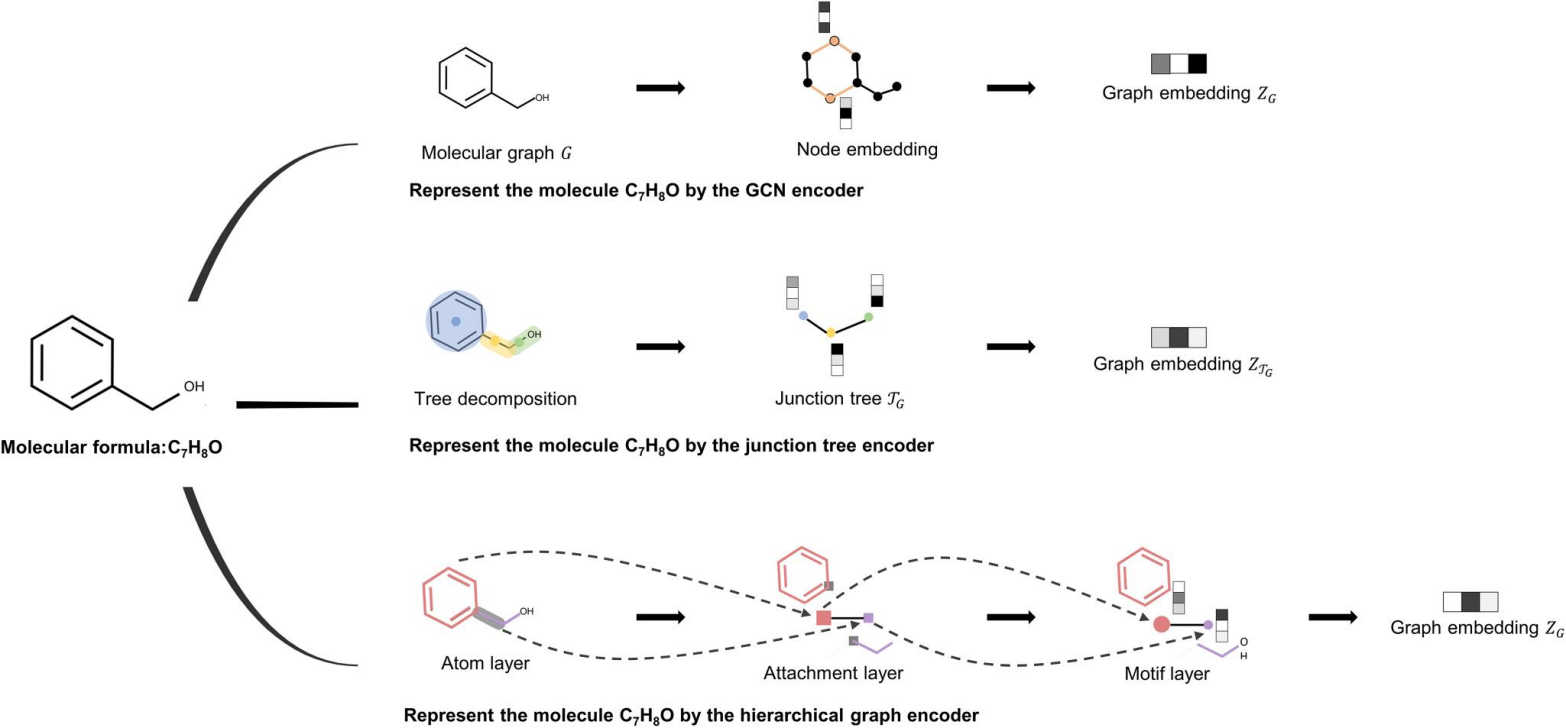
The molecule C_7_H_8_O is represented by three kinds of graph‐based representations.

Graph‐based molecular representations model a molecule as a molecular graph *G* = (*V*, *E*), where *V* is the node set of the graph mapping to atoms and *E* denotes the edge set of the graph mapping to chemical bonds [43]. Moreover, nodes may be assigned features representing the properties of atoms. Graph‐based molecular representations consist of two parts: node embedding and graph embedding. The node embedding is the most important one of them, as it can be aggregated into the graph embedding. To learn the molecular graph representations, researchers utilized different graph neural network (GNN) encoders, which usually follow the message‐passing neural network (MPNN) framework. Assouel et al. [44] used a graph convolution network (GCN) encoder for molecular optimization, Jin et al. [45] proposed a junction tree encoder with a tree message‐passing network, and Jin et al. [46] designed a hierarchical graph encoder by a hierarchical message‐passing network. Among these encoders, the GCN encoder first performs several spatial graph convolutions on the input graph and then aggregates the node embeddings into a single graph latent representation. Using convolution operations, this GCN encoder can capture the whole topology information of the graph. By extracting certain clusters (such as rings and bonds) into a single node, the junction tree encoder first decomposes a molecular graph *G* into a junction tree $T_{G}$, and then obtains the representation of $T_{G}$ by aggregating the node information. The junction tree encoder provides a reliable cluster vocabulary to extract substructures ensuring generating valid molecules. Moreover, the hierarchical graph encoder contains three MPNN layers named the atom layer, attachment layer, and motif layer, respectively; after updating embeddings through these layers, the graph embedding is finally aggregated. Due to learning a hierarchical representation, the hierarchical graph encoder can obtain both coarse‐grained motif and fine‐grained atom connectivity. As shown in Figure 2, a molecule can be represented by the above three kinds of graph‐based representations.

## 3 METHODS

**FIGURE 3:**
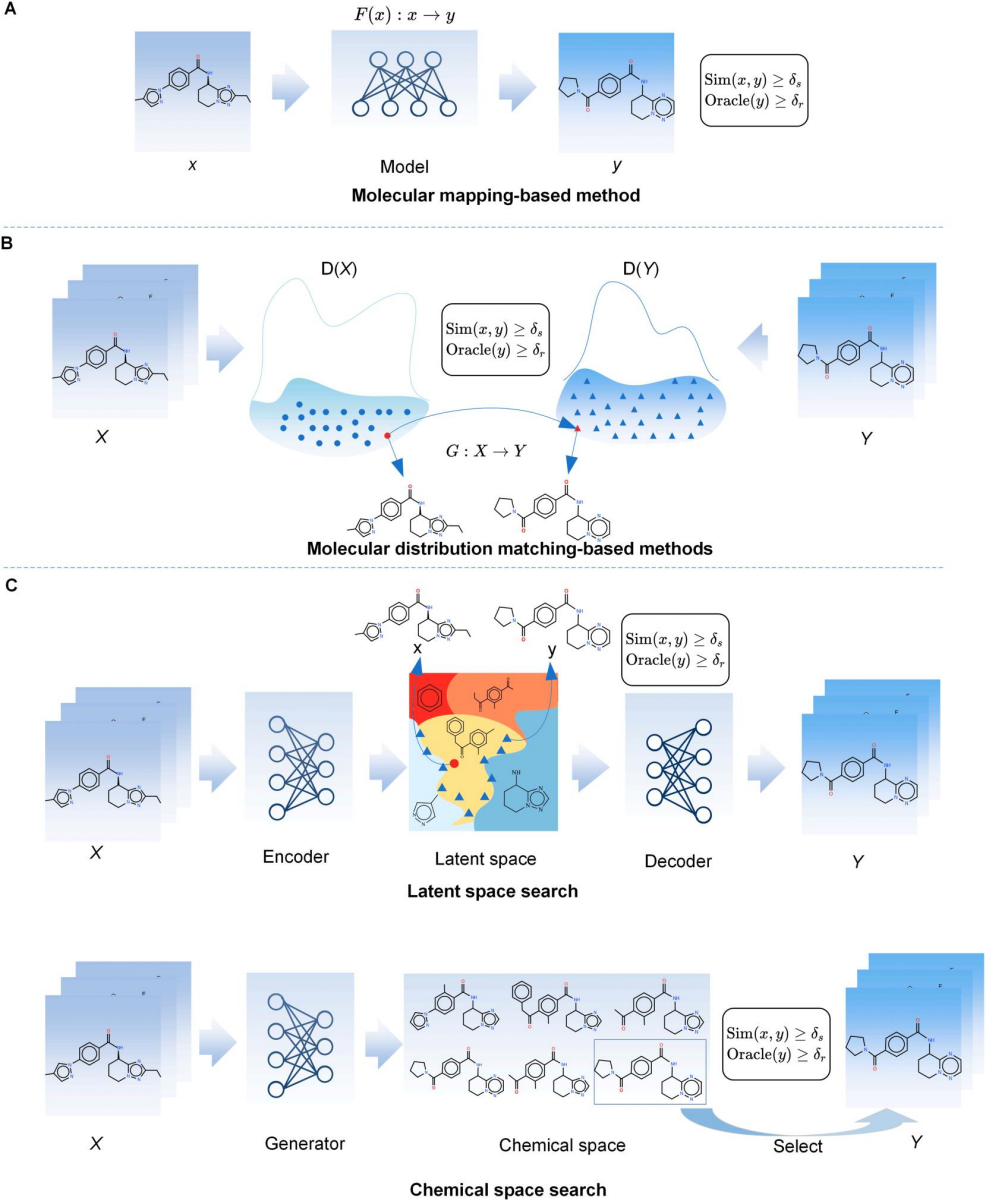
Artificial intelligence‐based molecular optimization methods.

**FIGURE 4:**
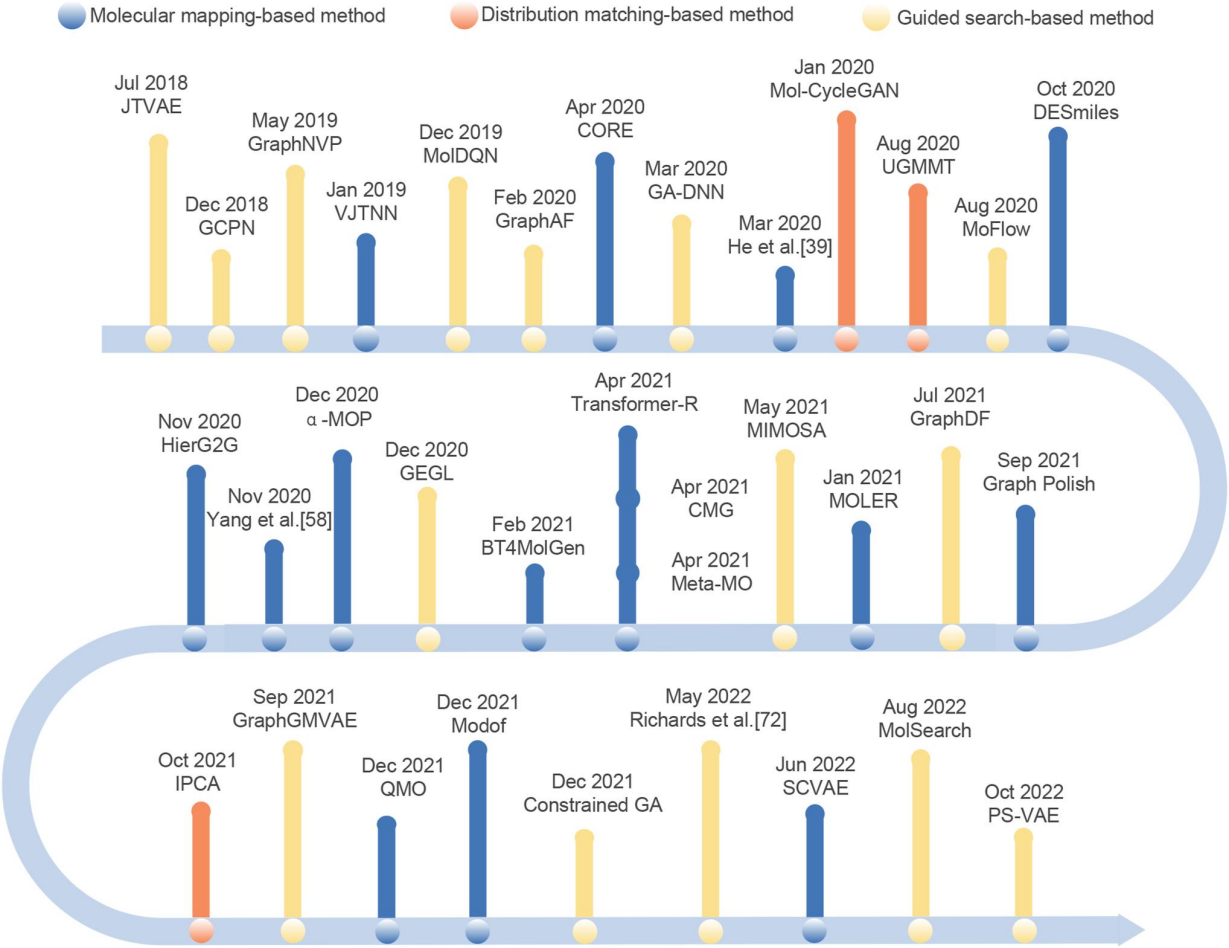
The timeline of molecular optimization models.

In recent years, with the development of Big Data and AI technologies, great attempts have been made to use AI technologies, especially deep learning, to automatically learn domain knowledge from large amounts of data to guide molecular optimization. As shown in Figure 3, the molecular optimization methods are roughly classified into three types: molecular mapping‐based method, distribution matching‐based method, and guided search‐based method. Figure 4 shows the timeline of the molecular optimization model based on these three methods.

### 3.1 Molecular mapping‐based method

Molecular mapping is to learn the mapping relationship *F* between matched molecular pairs (MMPs). Given an MMP (*x*, *y*), where *x* and *y* have small differences between consecutive atoms but huge differences between properties, the molecular mapping can be formally represented as *F*(*x*): *x*→*y*. Molecular mapping‐based method is implemented by medicinal chemistry transformation rules learned from MMPs, which imitates the knowledge and experience of medicinal chemists. Based on the molecular representation, there are two types of molecular mapping‐based methods, including 1D string representation‐based Sequence to Sequence methods and 2D molecular graph representation‐based Graph to Graph methods. The details of publicly available MMPs datasets are shown in Supplementary Table S1.

#### 3.1.1 Sequence to sequence

The molecular optimization methods of Sequence to Sequence are usually modeled as a sequence translation problem with the help of methods in natural language processing (NLP). He et al. [39] constructed the model based on the transformer architecture for property variations between MMPs, using the source molecules and property constraints (the true and desired property scores of source molecules) as inputs to the model, and trained the model to output the target molecules. He et al. [47] later extended their model and proposed Transformer‐R. Transformer‐R cuts the SMILES of the source molecule into a core SMILES representation and an R‐group SMILES representation, separated by using a separator. Instead of generating the whole molecule, the transformer generates the R‐group. To solve the multi‐property optimization problem, Shin et al. [48] designed a new transformer architecture‐based model named CMG. CMG introduced the property prediction and similarity prediction regularization network in the architecture and proposed an improved beam search algorithm to facilitate better utilization of the auxiliary network. In contrast to the SMILES representation, molecular fingerprints contain information about the molecular structure, while chemically similar molecules usually have similar fingerprints. Maragakis et al. [49] proposed a model based on RNN architecture named DESMILES. DESMILES used molecular fingerprints as the input for the deep learning model to transform the source molecular fingerprints into their associated SMILES molecules. The molecular fingerprint to SMILES transformation relationship is pre‐trained on a large dataset, and then the model is fine‐tuned using matched molecular pairs.

#### 3.1.2 Graph to graph

The molecular optimization methods of Graph to Graph are usually modeled as a graph translation problem similar to image‐to‐image translation in the image domain. Jin et al. [50] first proposed to describe the molecular optimization problem as a supervised graph‐to‐graph translation problem and extended the JTVAE architecture to design the VJTNN model. VJTNN learned the difference vector of molecular pairs while using this vector and the input molecule information to decode the optimized molecules during the decoding process. The attention mechanism is presented in the tree decoding process and combined with adversarial training to avoid invalid translation. Jin et al. [46] further extended their work by proposing a hierarchical graph VAE named HierG2G. HierG2G used a larger and more flexible motif instead of the smaller substructure in the JTVAE. The decoding process sequentially predicts whether to add substructures, type of substructure, and attachment points of the substructure and their corresponding attachment points in the current graph. This decoding strategy allows modeling the strong dependency between the process of successive addition and selection of substructure and significantly improves the accuracy of reconstruction and generation. Fu et al. [20] designed the copy & refine strategy named CORE based on VJTNN. CORE predicted the probability of copying a substructure in the process of decoding molecules, and then further predicts which specific substructure should be used. Thus, CORE solved the problem that the decoding process involves complex combinatorial enumeration in the process of connecting substructure prediction, which leads to a slow and difficult to parallelize decoding process. Yu et al. [51] introduced structural constraints based on VJTNN and proposed a structure‐aware conditional variational autoencoder named SCVAE. SCVAE used the topology of molecules as structural conditions to optimize the properties of molecules through constrained structural modifications. Fu et al. [52] proposed a molecule‐level reward function named MOLER. MOLER introduced the similarity reward and size deviation penalty to the architecture of VJTNN to enhance the structural similarity constraint, meanwhile preventing excessive size deviation between the source and target molecules. Ji et al. [53] proposed a teacher and student polish named T&S polish architecture. T&S polish aimed to reduce the generation process of the target molecule by capturing more useful information from the source molecule. The teacher component automatically identifies and annotates the optimization centers and the saving, removal, and addition of certain parts of the molecule. The student component uses this knowledge learned by the teacher component and applies it to the new molecule. Similar to T&S polish, the work of Chen et al. [54] proposed a one‐fragment‐based optimization model named Modof. Modof encoded the difference between molecules before and after optimization at a breakpoint, predicts the location of a single broken chain in the source molecule from a vector sampled in the potential difference space, and modifies the molecule by changing the fragment at that location. Since there may be multiple ways to optimize a molecule, the authors introduce an iterative optimization strategy to extend Modof to Modof‐pipem, which modifies a given molecule into multiple optimized molecules.

Molecular mapping‐based methods require a large number of MMPs, however, such data are not easy to obtain and require some heuristic algorithms [55, 56] to construct, while the model performance of such methods depends heavily on the quality of MMPs. Researches have been done to address molecular mapping‐based method under conditions of sparse data. Yang et al. [57] proposed an efficient self‐training method based on pretrained molecular generation models and property predictors to expand the training data by generating additional data satisfying the constraints during the iterative training process. Inspired by the back translation in NLP, Fan et al. [58] proposed a semi‐supervised method named BT4MolGen. BT4MolGen trained the pseudo‐labeled data generation model with MMPs, and then trained the forward molecular optimization model using the pseudo‐labeled and labeled data, which can utilize a large amount of unlabeled data and alleviate the data sparsity problem. Wang et al. [59] proposed a first‐order meta‐learning algorithm named Meta‐MO for molecular optimization. Meta‐MO enabled the model to be adapted to few‐shot molecular optimization tasks. Fu et al. [60] proposed a method named *α*‐MOP to unify the molecular mapping‐based method and Reinforcement learning (RL) using *α*‐divergence. By controlling the *α* value in the objective function, the weights of the molecular mapping‐based method and the RL target are dynamically adjusted. In the early stage, more attention is paid to the molecular mapping‐based method learning target, while more learning weights are gradually shifted to the RL target in the later stage.

### 3.2 Molecular distribution matching‐based methods

Drawing on the idea of style transfer in the image field, molecular distribution matching is to learn the mapping relationship *G* between the matched molecular set (MMS) rather than the matched molecular pairs of MMPs. Given an MMS (*X*, *Y*), where *X* and *Y* have huge differences between properties, molecular distribution matching can be formally represented as *G*:*X* → *Y*. The molecular optimization is achieved by adjusting the distribution of chemical spatial properties of the molecules in *X* to be close to the distribution of *Y*.

Maziarka et al. [61] designed a molecular optimization model named Mol‐CycleGAN based on the Cycle‐GAN model. Firstly, Mol‐CycleGAN used JTVAE to represent each molecule as a point in the latent space, by learning the latent space representation distribution of two sets of MMS. After that, this model used the Cycle‐GAN model to learn the universal mapping relationship from the source molecular set to the target molecular set. Barshatski et al. [40] proposed an end‐to‐end dual learning method named UGMMT to train a bidirectional translation of the source molecular set to the target molecular set embedding space under the double cycle constraints, and incorporated a molecular attention mechanism to drive the generation of molecules to maintain similarity with the chemical fingerprint (FP) of the source molecule. Later, Barshatski et al. [62] extended UGMMT and proposed a multi‐property optimization method named IPCA. IPCA learns one transformation for each property optimization while constraining the latent embedding space between all the transformations, and proposed a new adaptive loss to balance the separated transformations while stabilizing the optimization process.

### 3.3 Guided search‐based methods

Guided search‐based methods search for target molecules either in the chemical space of molecules or in a latent space learned by encoder‐decoder models, guided by the molecular property predictor or statistical models [63]. The latent space search and chemical space search are briefly introduced below.

#### 3.3.1 Latent space search

The latent space search first encodes the source molecule into latent embedding in a low‐dimensional latent space, then finds latent embeddings that satisfy constraints by searching the region around source molecules in the latent space, and finally decodes them back into the chemical space. In most cases, a property predictor is necessary to guide the search in the latent space.

Some latent space search methods have to train property predictors in the latent space to guide research. Jin et al. [45] proposed a junction tree variational autoencoder named JTVAE. JTVAE first decomposed a molecular graph into the junction tree with each node representing a molecular substructure, and then mapped both the junction tree and molecular graph into the latent embeddings. Meanwhile, JTVAE trained a property score predictor in the latent space, to ensure the distribution of molecules in the latent space will be clustered together according to the property values. The gradient ascent is employed in the latent space to search latent embeddings with improved property scores. Zang et al. [64] proposed a flow‐based graph generation model named MoFlow. MoFlow first generated bonds of molecules based on the Glow model [65], then used the designed graph conditional flow framework to generate atoms based on the given bonds, and finally assembled them into a valid molecular graph. MoFlow also applied gradient ascent to find the latent embeddings with better property scores. Kong et al. [66] proposed Principal Subgraph Variational Auto‐Encoder named PS‐VAE. Specifically, the source molecules are decomposed into principal subgraphs according to the designed principal subgraph vocabulary, while the principal subgraphs are encoded into the latent space using GNN. After that, the sequence of principal subgraphs is autoregressively decoded from the latent embeddings optimized through gradient ascent by GRU, and the sequence of principal subgraphs is concatenated into a complete graph using a link prediction method. Some methods adopt other optimization algorithms to search in the latent space. Madhawa et al. [67] proposed a normalizing flow (real NVP)‐based molecular graph generation model named GraphNVP [68]. GraphNVP designed two types of reversible affine coupling layers to convert the adjacency tensor and node feature matrix of the molecular graph into latent embeddings, respectively. In the latent space, the randomly selected latent embeddings are interpolated to obtain embeddings with desired properties based on the trained property predictor.

There have been lots of state‐of‐the‐art property predictors such as Oliverona et al.’s method [69] and RDkit, and some researchers directly adopted these property predictors instead of training their own predictors. Hoffman et al. [63] proposed a query‐based molecular optimization framework named QMO. QMO utilized the pretraining encoder and decoder to learn molecular embeddings, and randomly sampled the neighboring embeddings of the source molecule in the latent space, evaluated the properties and structural similarity constraint of the corresponding decoded molecules with Oliverona et al.’s method and RDkit, and performed a zero‐order function to estimate the gradient thus finding better embeddings using gradient descent, and progressively optimizes the input source molecules by evaluating the properties of the generated molecules. Richards et al. [70] mapped source molecules into the latent space using *β*‐VAE, and selected the neighbors around the embedding of the source molecules based on standard Gaussian sampling and used them as the initial particles of the particle swarm optimization (PSO) algorithm. The fitness of the decoded molecule is evaluated through a reward function containing property constraint and structural similarity constraint.

Besides, there are some latent space search methods free of property predictors. Yu et al. [71] proposed the Generative Scaffold Hopping Model named GraphGMVAE. GraphGMVAE used Dual‐MPNN [72] to transform the source molecule and its scaffold into the corresponding scaffold embedding and side chain embedding, respectively. After that, GraphGMVAE sampled the new scaffold embedding from the Gaussian mixture distribution in the latent scaffold space with different hopping rates, and the new scaffold embedding is fed to the GRU decoder with the original side chain embedding and property conditions, to produce molecules with the new scaffold and bioactivity condition.

#### 3.3.2 Chemical space search

In contrast to the latent space search, the chemical space search methods search for molecules that satisfy constraints in the high‐dimensional, discrete chemical space directly. Many advanced optimization algorithms, such as reinforcement learning and genetic algorithm, have been used in the chemical space search.

RL consists of three main components: agent, reward function, and the environment. The agent selects the action based on the state, and the environment evaluates the quality of the action based on the reward function and provides feedback to the agent. In molecular optimization, the state generally denotes a partially generated molecule, actions are the modification operation for the molecule, and reward function involves desired properties reward and structural similarity constraint reward. You et al. [73] designed a graph convolutional policy network named GCPN. GCPN adopted a stepwise method for molecular graph generation, formulating the problem as a Markov Decision Process (MDP) [74] to train the agent in a chemistry‐aware environment. GCPN applied two types of rewards to guide the agent, one is a validity reward, which encourages the chemical valence rules obeyed during stepwise generation, and the other is an adversarial reward that ensures the similarity between the source molecule and the generated molecule. Zhou et al. [75] proposed MolDQN model combining RL with chemical rules to ensure 100% chemical validity of molecules, where the modification of molecules is described as the MDP and uses the deep Q‐networks [76] to solve this MDP with the desired properties and structural similarity constraint as rewards. Molecular modification is achieved by a series of actions including atom addition, bond addition, and bond removal with the guidance of reward functions.

Another molecular optimization method is to first pretrain the molecular generation model and then fine‐tune the molecular generation process with RL, such as GraphAF [77] and GraphDF [78]. GrpahAF designed an autoregressive flow‐based molecular generation model that adopts a sequential molecular graph generation strategy, which adds an atom or a bond and guarantees validity via incorporating a valency check in each step. GraphDF was a novel discrete latent variable model for molecular graph generation based on normalizing flow methods and used invertible modulo‐shift transforms to map discrete latent variables to nodes and edges of the graph. In the fine‐tuning stage, GraphAF and GraphDF used the randomly sampled subgraph of the source molecule as the initial state and the molecular generative model as the environment, which used a reward function similar to GCPN and proximal policy optimization (PPO) algorithm to fine‐tune the generative model.

Genetic algorithm (GA) is a popular population optimization algorithm inspired by the concepts of biological evolution and natural selection. GA selects molecules from initial populations according to the fitness function, applies the crossover (e.g., swap functional groups between two molecules) and mutation (e.g., switch an atom type, delete an atom, or add an atom) operations on them to generate the new population, and then repeats the aforementioned process until achieving the termination condition. Specifically, in the phase of selection, molecules with higher fitness scores are more likely to be selected, and the roulette algorithm is a common method of selection. Nigam et al. [79] proposed a discriminator‐based molecular graph generation model named GA‐DNN. GA‐DNN used a linear combination of the molecular property score and the discriminator score as the fitness function, and then selects molecules by a smooth logistic function. After that, GA‐DNN applied the random mutations of the selected molecules, while there is no crossover operation because character deletion is implicitly taken into account in the mutation. Specifically, in the molecular optimization process, GA‐DNN used the combination of the molecular property score and the structural similarity constraint as the fitness function, without the discriminator score. Lee et al. [52] introduced a novel GA method named constrained GA. Constrained GA also used the combination of the molecular property score and the structural similarity constraint as the fitness function, and then selects molecules by a smooth logistic function. After that, constrained GA applied an operator of graph‐based crossover presented in GB‐GA [80] and an operator of SELFIES‐based mutation (e.g., tom replacement, atom insert, and atom deletion) to create new molecules. In addition, to make sure the algorithm can always search for feasible solutions, a single structural similarity constraint is used before the selection operator.

Ahn et al. [81] developed a genetic expert‐guided learning method named GEGL containing the expert strategy and the apprentice strategy. The expert strategy is employed to modify the molecules in the max‐reward priority queues generated by the apprentice strategy (molecule‐generating DNN) through domain‐specific genetic operators (mutation and crossover). The apprentice strategy tuned its parameters by imitating the high‐reward molecules found by the expert strategy through imitative learning. GEGL applied the framework to the molecular optimization task by initializing the maximum reward priority queue as the source molecule and adding structural similarity constraints to the reward function. In addition to RL and GA, some researchers have tried other methods for molecular optimization. Sun et al. [82] proposed a multi‐property molecular optimization method named MolSearch combining Monte Carlo tree search (MCTS) with a multi‐objective optimization algorithm. MolSearch divided the search process into two stages, the first stage optimizes biological properties and the second stage optimizes non‐biological properties. Starting from the source molecule, MolSearch modifies the molecule based on predefined modification actions [38], retains the Pareto optimality solution in each iteration, and screens the molecules that meet the requirements from the Pareto set of molecules at the end of the iterative search. Fu et al. [83] proposed a new multi‐property molecular optimization framework named MIMOSA based on Markov Chain Monte Carlo (MCMC) sampling. MIMOSA used a pretrained GNN to generate new candidate molecules and associated weights, followed by sampling from the target distribution using MCMC.

According to the above content, molecular mapping‐based methods use MMPs as training data and usually perform better due to the explicit guidance of the target molecule. However, such molecular data are not always easy to obtain, and the performance of the model depends heavily on the quality of the constructed molecular pairs. The advantage of performing optimization in the latent space is that the latent space learned by the model is usually continuous and low‐dimensional, which makes the problem in the optimization phase easier to solve, and avoids the combinatorial search of high‐dimensional discrete molecular data structures. Moreover, regularization or structural priors can be easily imposed when learning the latent space, especially for highly discrete, high‐dimensional, and non‐differentiable molecular data. The advantage of the chemical space search strategy can combine domain knowledge in the search process. The latent space search requires the encoding and decoding process of molecules, while the molecular chemical space search is done directly in the molecular chemical space to avoid the loss of information to a certain extent. Guided search‐based methods usually have the disadvantages of low efficiency and long search time due to the lack of target molecular information guidance. From the data perspective, distribution matching‐based methods are intermediate between the molecular mapping‐based methods and the guided search‐based methods. The information of the target molecular domain provided by the target molecular set is used to improve the optimization efficiency, while avoiding the model reliance on such difficult‐to‐obtain MMPs data. As shown in Supplementary Tables S2–S5, we summarize the results of molecular optimization on four datasets provided by Jin et al. [50].

### 3.4 Evaluation metrics

Model evaluation is an important task of the molecular optimization task. Based on the training molecular set S, after building the pilot optimization model, the model is used to optimize the test molecular set *M* to obtain the optimized effective (i.e., satisfying the basic chemistry rules) molecular set *M* ^′^. The following metrics are often used to evaluate the performance of the optimization model for the molecular sets *M* and *M* ^′^ before and after optimization.

**Similarity**. The similarity metric measures the average similarity between the set of molecules *M* and *M* ^′^ and is obtained by calculating the Tanimoto similarity for each pair (*m*, *m* ^′^), *m* ∈ *M*, *m* ^′^∈ *M* ^′^ of molecules before and after optimization. The calculation formula is as follows:

$$\text{Sim}\left(M,M^{\prime }\right)=\frac{1}{\left|M\right|}\sum \frac{\left|f_{m}⋂f_{m^{\prime }}\right|}{\left|f_{m}⋃f_{m^{\prime }}\right|}$$

where *f*_*m*_ and $f_{m^{\prime }}$ denote the Morgan fingerprints [84] of molecules *m* and *m* ^′^, respectively.

**Diversity**. The diversity metric measures the average difference between the set of molecules *M* and *M* ^′^, and is obtained by calculating the Tanimoto distance for each pair of molecular pairs (*m*, *m* ^′^):

$$\text{Diversity}\left(M,M^{\prime }\right)=\frac{1}{\left|M\right|}\sum (1-\frac{\left|f_{m}⋂f_{m^{\prime }}\right|}{\left|f_{m}⋃f_{m^{\prime }}\right|})$$

**Novelty**. Novelty metric measures the ratio of new molecules in the optimized set of molecules *M* ^′^, obtained by counting the number of optimized molecules that never appear in the training set of molecules *S*, and is calculated as follows:

$$\text{Novelty}\left(M^{\prime }\right)=1-\frac{\left|M^{\prime }⋂S\right|}{\left|S\right|}$$

**Optimized property score**. The optimized property score metric measures the average property score of the optimized molecular set *M* ^′^, obtained by calculating the property score of each optimized molecule *m* ^′^:

$$\text{OPS}\left(M^{\prime }\right)=\frac{1}{\left|M^{\prime }\right|}\sum \text{Oracle}\left(m^{\prime }\right)$$

where Oracle(⋅) is a function that calculates the score of the molecular property.

**Improvement**. The average property improvement metric measures the average property score difference between the molecular sets *M* and *M* ^′^, and is obtained by calculating the property score difference for each pair of molecular pairs (*m*, *m* ^′^):

$$\text{Improvement}\left({M,M}^{\prime }\right)=\frac{1}{\left|M\right|}\sum \left(\text{Oracle}\left(m^{\prime }\right)-\text{Oracle}\left(m\right)\right)$$

**Success**. The success rate metric measures the ratio of molecules in the optimized set of molecules *M* ^′^ that satisfy both the similarity metric threshold *δ*_*s*_ and the property threshold *δ*_*r*_. Based on different kinds of properties, there are two definitions of the success rate metric. For several properties such as log P, researchers wish the difference of property scores between molecules before and after optimization to be higher than *δ*_*r*_, while for other properties such as QED, researchers just wish the property scores of molecules after optimization to be higher than *δ*_*r*_, which are calculated as follows:

$$\text{Success}\left(M^{\prime }\right)=\frac{|\left\{m^{\prime }\right.|\text{Sim}\left(m,m^{\prime }\right)\geq \delta_{s},\text{Oracle}\left(m^{\prime }\right)-\text{Oracle}\left(m\right)\geq \delta_{r},m^{\prime }\notin S\}|}{\left|M^{\prime }\right|}$$

or

$$\text{Success}\left(M^{\prime }\right)=\frac{|\left\{m^{\prime }\right.|\text{Sim}\left(m,m^{\prime }\right)\geq \delta_{s},\text{Oracle}\left(m^{\prime }\right)\geq \delta_{r},m^{\prime }\notin S\}|}{\left|M^{\prime }\right|}$$

We provide performance comparisons of the existing molecular optimization methods on publicly available datasets in the Appendix.

## 4 DISCUSSION

Despite the significance of developing deep learning methods for practical drug discovery tasks, molecular optimization is technically complex to accomplish due to several major challenges.

The first challenge is the limited number of molecular datasets and properties for molecular optimization. For molecular optimization, researchers usually compile the MMP dataset and MMS datasets from molecular property datasets. Although the molecular property datasets include a huge number of molecules for some properties, there is a scarcity of labeled data for other desired properties to be optimized, such as toxicity and bioactivity. As described in Section 2.3, MMP datasets collect source and target molecules, while MMS datasets rely on the collection of two molecular sets with huge differences between properties, and thus it is difficult to compile the MMP datasets and MMS datasets for all desired properties owing to the limitations in data availability. Although the latent space search methods can directly optimize the molecules without MMP datasets and MMS datasets, they utilize a high‐accuracy property predictor for guiding research, and it also needs sufficient molecules with labeled desired properties for training the property predictor. To overcome these data challenges, transfer learning and few‐shot learning strategies are necessary for molecular optimizations.

Secondly, great attention has been drawn to the interpretability and transferability of AI‐based methods [85, 86], which bring a new challenge to molecular optimization. In general, the optimization methods and the optimized molecules are difficult to understand by medicinal chemists, which indicates the lack of sufficient interpretability of the molecular optimization methods [87, 88]. The crucial point of interpretability in molecular optimization lies in identifying the substructures of molecules relevant to the desired properties and contributing to the optimization process. Recent research has highlighted transparency, justification, and informativeness as pivotal factors for model interpretability [89]. To address the interpretability challenge in optimization methods, the uncertainty estimation and feature attribution strategies are necessary to enhance interpretability of optimization methods. In addition, it is difficult to achieve effective results when optimizing the molecules outside the training datasets, which shows the transferability challenge for the optimization methods [90]. The pivotal point of transferability in molecular optimization is leveraging existing, transferable knowledge from related molecular tasks to enable the learning of separate optimization tasks with limited availability of molecular data. For the model transferability, there are four categories of research direction, that is, instance‐based, feature‐based, parameter‐based, and relation‐based approaches [91]. To tackle the transferability challenge in optimization methods, it is necessary to incorporate domain adaptation techniques as well as pretraining and fine‐tuning strategies in the molecular optimization task.

Thirdly, a challenging direction for molecular optimization is to develop optimization methods that can effectively optimize multiple properties simultaneously, aiming for global optimality. In general, most existing molecular optimization methods are to optimize single‐property of molecules. Recently, researchers have made efforts to the multi‐property optimization methods [92, 93], which exhibit greater complexity than single‐property optimization methods. On one hand, the multi‐property optimization is also limited by the insufficient data labeled with multi‐property; on the other hand, the desired properties for molecular optimization might contain a potential contradiction [10], and the improvement of some properties may lead to the deterioration of others, how to determine the tradeoff between different properties need to be resolved. Therefore, there is a pressing need to develop novel and efficient algorithms specifically for multi‐property optimization.

## 5 CONCLUSION

This study provides a comprehensive review of existing methods for molecular optimization. Additionally, characteristics of the molecular representation are presented, along with an introduction to the assessing criteria of the molecular optimization methods. In particular, a detailed introduction and comparison of the molecular optimization methods are conducted. This review points out the potential challenges as well as the exciting new prospects for molecular optimization, which will guide researchers who are interested in artificial intelligence molecular optimization.

## AUTHOR CONTRIBUTIONS

Yuhang Xia: Investigation; visualization; writing – original draft. Yongkang Wang: Investigation; writing – original draft. Zhiwei Wang: Investigation; visualization; writing – original draft. Wen Zhang: Conceptualization; funding acquisition; supervision; review.

## CONFLICT OF INTEREST STATEMENT

The authors Yuhang Xia, Yongkang Wang, Zhiwei Wang, and Wen Zhang declare no conflicts of interest.

## ETHICS STATEMENT

This is a review article and does not involve any research related to human or animal subjects.

## Supporting information

Supporting Information S1
