# Supplementary material for: A comprehensive review of molecular optimization in artificial intelligence‐based drug discovery
Source: Quant Biol. 2024 Feb 12;12(1):15–29. doi: 10.1002/qub2.30 (PMC12806414; doi:10.1002/qub2.30)
Supplement: Supplementary file 1 — Supporting Information S1 [file QUB2-12-15-s001.docx]

SUPPLEMENTARY MATERIALS

To enable comparison and statistical analysis, we just summary studies employing publicly available datasets for molecular optimization experiments. Specifically, for the Guided Search methods (GS), they used the ZINC-250K dataset, while for the Molecular Mapping-bathed methods (MM) and Distribution Matching-based methods (DM), they utilized four datasets (Table S1) provided by Jin et al.[1], which for the optimization of LogP04 (Table S2), LogP06 (Table S3), QED (Table S4) and bioactivity against DRD2 (Table S5), respectively. It is worth noting that, due to the limited number of studies on multi-property molecular optimization[2–5] and the lack of standardized datasets, We did not conduct a collective analysis of studies within this domain. However, interested readers may refer to the original publications for further details.

Table S1. Publicly available MMPs dataset information.

| Desired property | #Training | #Validation | #Test | $\boldsymbol{\delta}_{\boldsymbol{s}}$ | $\boldsymbol{\lambda}_{\boldsymbol{1}}$ | $\boldsymbol{\lambda}_{\boldsymbol{2}}$ |
| --- | --- | --- | --- | --- | --- | --- |
| LogP04 | 99909 | 200 | 800 | 0.4 | - | - |
| LogP06 | 75248 | 200 | 800 | 0.6 | - | - |
| QED | 88306 | 360 | 800 | 0.4 | [0.7,0.8] | [0.9,1.0] |
| Bioactivity against DRD2 | 34404 | 500 | 1000 | 0.4 | [0,0.05] | [0.5,1.0] |

Note: $\delta_{s}$ measures the similarity between the source and target molecules; $\lambda_{1}$ and $\lambda_{2}$ indicate the property range of source molecules and target molecules. The symbol ‘-’ indicates that there is no restriction on the property range.

Table S2. The results of Molecular optimization on the LogP04 dataset. All results are from the original paper.

|  | Methods | | Improvement | Similarity | Success(%)  ($\boldsymbol{\delta}_{\boldsymbol{s}}\boldsymbol{,}\boldsymbol{\delta}_{\boldsymbol{r}}$) | Diversity | OPS |
| --- | --- | --- | --- | --- | --- | --- | --- |
| MM | VJTNN | | 3.55 ± 1.67 | - | - | 0.480 | - |
|  | CORE | | - | 0.3695 | 56.47^#^  (0.4,0.8) | - | 3.1053 |
|  |  |  | - | 0.3695 | 27.88^#^  (0.4,1.2) | - | 3.1053 |
|  | DESMILES | | 3.04 ± 1.36 | - | - | 0.408 | - |
|  | HierG2G | | 3.98 ± 1.46 | - | - | 0.564 | - |
|  | BT4MolGen | | 4.41 | - | - | - | - |
|  | CMG | | 3.92 ± 1.88 | - | - | 0.545 | - |
|  | MOLER | CORE | 3.199 | 0.371 | 57.93^#^  (0.4,0.8) | - | - |
|  |  | VJTNN | 3.182 | 0.372 | 57.01^#^  (0.4,0.8) | - | - |
|  |  | JTVAE | 3.015 | 0.314 | 45.24^#^  (0.4,0.8) | - | - |
|  | α-MOP | | 3.0282 | 0.3620 | 56.43^#^  (0.4,0.8) | - | - |
|  | T&S Polish | | - | - | 42.25^#^  (0.3,0.8) | - | - |
|  |  |  | - | - | 38.75^#^  (0.4,1.2) | - | - |
|  |  |  | - | - | 43.14^#^  (0.4,0.6) | - | - |
|  | Modof | | 5.89±1.57 | 0.46±0.06 | - | - | - |
|  | SCVAE | | 3.95 ± 1.46 | - | - | 0.512 | - |
| DM | MolCycleGAN | | 2.89 ± 2.08 | 0.52 ± 0.10 | 58.75^*^ | - | - |
| GS | JTVAE | | 0.84 ± 1.45 | 0.51 ± 0.10 | 83.60^*^ | - | - |
|  | GCPN | | 2.49 ± 1.30 | 0.47 ± 0.08 | 100.0^*^ | - | - |
|  | MolDQN | | 3.37±1.62 | - | 100.0^*^ | - | - |
|  | GraphAF | | 8.21 ± 6.51 | 0.49 ± 0.09 | 99.88^*^ | - | - |
|  | GA-DNN | | 5.93 ± 1.41 | - | 100.0^*^ | - | - |
|  | MolFlow | | 4.71 ± 4.55 | 0.61 ± 0.18 | 85.75^*^ | - | - |
|  | GEGL | | 7.87 ± 1.81 | - | 100.0^*^ | - | - |
|  | GraphDF | | 9.19±6.43 | 0.48±0.08 | 99.63^*^ | - | - |
|  | QMO | | 7.71±5.65 | - | - | - | - |
|  | Constrained GA | | 5.53±1.29 | - | 100.0^*^ | - | - |
|  | Richards et al. | | 5.67 ± 2.05 | 0.42 ± 0.05 | 98.25^*^ | - | - |
|  | PS-VAE | | 4.19±1.30 | - | 98.9^*^ | - | - |

Note: The symbol ‘-’ indicates that no results exist from the original paper. The symbol ‘#’ and ‘^*^’ indicates the success rate is calculated differently. The success rate with superscript ‘^*^’ indicates $Sim\left( m,m^{'} \right)\geq\delta_{s}=0.4,\mathrm{Oracle}\left( m^{'} \right)-\mathrm{Oracle}\left( m \right)\geq\delta_{r}=0.$ In the success rate with superscript ‘#’, ($\delta_{s},\delta_{r}$) indicates $Sim\left( m,m^{'} \right)\geq\delta_{s},\mathrm{Oracle}\left( m^{'} \right)\geq\delta_{r}$. MM indicates **M**olecular **M**apping-based methods. MDM indicates **M**olecular **D**istribution **M**atching-based methods. GS indicates **G**uided **S**earch-based methods.

Table S3. The results of different molecular optimization methods on the LogP06 dataset. All results are from the original paper.

|  | Methods | Improvement | Similarity | Success (%)  ($\boldsymbol{\delta}_{\boldsymbol{s}}\boldsymbol{,}\boldsymbol{\delta}_{\boldsymbol{r}}$) | Diversity | OPS |
| --- | --- | --- | --- | --- | --- | --- |
| MM | VJTNN | 2.33 ± 1.24 | - | - | 0.333 | - |
|  | CORE | - | 0.6386 | 57.64^#^  (0.4,0.8) | - | 2.021 |
|  |  | - | 0.6386 | 26.58^#^  (0.4,1.2) | - | 2.021 |
|  | DESMILES | 2.43 ± 1.11 | - | - | 0.299 | - |
|  | HierG2G | 2.49 ± 1.09 | - | - | 0.381 | - |
|  | BT4MolGen | 2.86 | - | - | - | - |
|  | T&S Polish | - | - | 86.63^#^  (0.3,0.8) | - | - |
|  |  | - | - | 83.37^#^  (0.4,1.2) | - | - |
|  |  | - | - | 88.54^#^  (0.4,0.6) | - | - |
|  | Modof | 3.14±1.77 | 0.65±0.05 | - | - | - |
|  | SCVAE | 2.66 ± 1.25 | - | - | 0.308 | - |
| DM | MolCycleGAN | 1.22 ± 1.48 | 0.69 ± 0.07 | - | 0.1925 | - |
| GS | JTVAE | 0.21 ± 0.71 | 0.69 ± 0.06 | 46.40^*^ | - | - |
|  | GCPN | 0.79 ± 0.63 | 0.68 ± 0.08 | 100.0^*^ | - | - |
|  | MolDQN | 1.86±1.21 | - | 100.0^*^ | - | - |
|  | GraphAF | 4.98 ± 6.49 | 0.66 ± 0.05 | 96.88^*^ | - | - |
|  | GA-DNN | 3.44 ± 1.09 | - | - | 0.998 | - |
|  | MolFlow | 2.10 ± 2.86 | 0.79 ± 0.14 | 58.25^*^ | - | - |
|  | GEGL | 4.43 ± 1.53 | - | 100.0^*^ | - | - |
|  | GraphDF | 4.51±5.80 | 0.65±0.05 | 92.13^*^ | - | - |
|  | QMO | 3.73±2.85 | - | - | - | - |
|  | Constrained GA | 3.67±1.29 | - | 100.0^*^ | - | - |
|  | Richards et al. | 5.67 ± 2.05 | 0.42 ± 0.05 | 100.0^*^ | - | - |
|  | PS-VAE | 2.52±1.12 | - | 100.0^*^ | - | - |

Note: The success rate with superscript ‘^*^’ indicates $Sim\left( m,m^{'} \right)\geq\delta_{s}=0.6,\mathrm{Oracle}\left( m^{'} \right)-\mathrm{Oracle}\left( m \right)\geq\delta_{r}=0.$ In the success rate with superscript ‘#’, ($\delta_{s},\delta_{r}$) indicates $Sim\left( m,m^{'} \right)\geq\delta_{s},\mathrm{Oracle}\left( m^{'} \right)\geq\delta_{r}$.

Table S4. The results of different molecular optimization methods on the QED dataset. All results are from the original paper.

|  | Methods | | Simila-rity | Diversity | Novelty | OPS | Improvement | Success (%)  ($\boldsymbol{\delta}_{\boldsymbol{s}}\boldsymbol{,}\boldsymbol{\delta}_{\boldsymbol{r}}$) |
| --- | --- | --- | --- | --- | --- | --- | --- | --- |
| MM | VJTNN | | - | 0.376 | 0.990 | - | - | 60.6  (0.4,0.9) |
|  | DESMILES | | - | 0.412 | - | - | - | 77.8  (0.4,0.9) |
|  | HierG2G | | - | 0.477 | - | - | - | 76.9  (0.4,0.9) |
|  | Yang et al. | | - | 0.514 | - | - | - | 93.1  (0.4,0.9) |
|  | BT4MolGen | | - | 0.390 | 99.5 | 0.829 | - | - |
|  | α-MOP | | 0.3264 | - | - | 0.9101 | - | 49.91(0.3,0.6) |
|  | Modof | | 0.48±0.07 | - | - | - | 0.18±0.03 | 66.25  (0.4,0.9) |
|  | SCVAE | | - | 0.423 | 0.986 | - | - | 58.1  (0.4,0.9) |
|  | CORE | | 0.3211 | - | - | 0.8952 | - | 50.26(0.3,0.6) |
|  |  |  | 0.3211 | - | - | 0.8952 | - | 27.23(0.4,0.8) |
|  | MO-LER | CORE | 0.360 | - | - | 0.910 |  | 57.32(0.3,0.6) |
|  |  | VJTNN | 0.351 | - | - | 0.904 | - | 56.32(0.3,0.6) |
|  |  | JTVAE | 0.302 | - | - | 0.858 | - | 43.20(0.3,0.6) |
|  | T&S Polish | | - | - | - | - | - | 69.38  (0.3,0.6) |
|  |  |  | - | - | - | - | - | 38.38  (0.4,0.8) |
|  |  |  | - | - | - | - | - | 22.53  (0.4,0.9) |
| DM | UGMMT | | 0.365 ± 0.003 | 1.000 ± 0.000 | 0.997 ± 0.001 | 0.855 ± 0.001 | - | 51.3±0.09  (0.3,0.8) |
| GS | QMO | | - | - | - | - | - | 92.8  (0.4,0.9) |

Table S5. The results of different molecular optimization methods on the DRD2 dataset. All results are from the original paper.

|  | Methods | | Simila-rity | Diversity | Novelty | OPS | Improvement | Success (%)  ($\boldsymbol{\delta}_{\boldsymbol{s}}\boldsymbol{,}\boldsymbol{\delta}_{\boldsymbol{r}}$) |
| --- | --- | --- | --- | --- | --- | --- | --- | --- |
| MM | VJTNN | | - | 0.162 | 0.827 | - | - | 78.4  (0.4,0.5) |
|  | DESMILES | | - | 0.328 | - | - | - | 96.8  (0.4,0.5) |
|  | HierG2G | | - | 0.192 | - | - | - | 85.9  (0.4,0.5) |
|  | Yang et al. | | - | 0.418 | - | - | - | 97.6  (0.4,0.5) |
|  | BT4MolGen | | - | 0.238 | 0.949 | 0.674 | - | - |
|  | α-MOP | | 0.3232 | - | - | 0.7928 | - | 49.32(0.3,0.6) |
|  | CORE | | 0.3334 | - | - | 0.7694 | - | 47.91  (0.4,0.8) |
|  |  |  | 0.3334 | - |  | 0.7694 | - | 17.31  (0.4,1.2) |
|  | Modof | | 0.46±0.05 | - | - | - | 0.88±0.12 | 88.60  (0.4,0.5) |
|  | MO-LER | CORE | 0.352 | - | - | 0.782 | - | 49.47  (0.3,0.6) |
|  |  | VJTNN | 0.334 | - | - | 0.778 | - | 47.39  (0.3,0.6) |
|  |  | JTVAE | 0.314 | - | - | 0.732 | - | 40.01  (0.3,0.6) |
|  | T&S Polish | | - | - | - | - | - | 54.54  (0.3,0.6) |
|  |  |  | - | - | - | - | - | 22.84  (0.4,0.8) |
|  |  |  | - | - | - | - | - | 38.41  (0.4,0.5) |
| DM | UGMMT | | 0.284 ± 0.001 | 0.914 ± 0.007 | 0.799 ± 0.009 | 0.826 ± 0.009 | - | 19.2±1.0  (0.3,0.8) |

REFERENCES

1. Jin W, Yang K, Barzilay R, Jaakkola T. Learning Multimodal Graph-to-Graph Translation for Molecule Optimization. 2019.

2. He J, You H, Sandström E, Nittinger E, Bjerrum EJ, Tyrchan C, et al. Molecular optimization by capturing chemist’s intuition using deep neural networks. J Cheminformatics. 2021;13:26.

3. Sun M, Xing J, Meng H, Wang H, Chen B, Zhou J. MolSearch: Search-based Multi-objective Molecular Generation and Property Optimization. In: Proceedings of the 28th ACM SIGKDD Conference on Knowledge Discovery and Data Mining. Washington DC USA: ACM; 2022. p. 4724–32.

4. Fu T, Xiao C, Li X, Glass LM, Sun J. MIMOSA: Multi-constraint Molecule Sampling for Molecule Optimization. Proc AAAI Conf Artif Intell. 2021;35:125–33.

5. Shin B, Park S, Bak J, Ho JC. Controlled molecule generator for optimizing multiple chemical properties. In: Proceedings of the Conference on Health, Inference, and Learning. New York, NY, USA: Association for Computing Machinery; 2021. p. 146–53.
